# The L1-dependant and Pol III transcribed Alu retrotransposon, from its discovery to innate immunity

**DOI:** 10.1007/s11033-021-06258-4

**Published:** 2021-03-16

**Authors:** Ludwig Stenz

**Affiliations:** 1 Department of Genetic Medicine and Development, Faculty of Medicine, Geneva University, Geneva, Switzerland; 2 Swiss Centre for Applied Human Toxicology, University of Basel, Basel, Switzerland

**Keywords:** Alu, Retrotransposons, DNA methylation, Reprogramming, Pluripotency, Imprinting, Mosaicism

## Abstract

The 300 bp dimeric repeats digestible by *Alu*I were discovered in 1979. Since then, Alu were involved in the most fundamental epigenetic mechanisms, namely reprogramming, pluripotency, imprinting and mosaicism. These Alu encode a family of retrotransposons transcribed by the RNA Pol III machinery, notably when the cytosines that constitute their sequences are de-methylated. Then, Alu hijack the functions of ORF2 encoded by another transposons named L1 during reverse transcription and integration into new sites. That mechanism functions as a complex genetic parasite able to copy-paste Alu sequences. Doing that, Alu have modified even the size of the human genome, as well as of other primate genomes, during 65 million years of co-evolution. Actually, one germline retro-transposition still occurs each 20 births. Thus, Alu continue to modify our human genome nowadays and were implicated in de novo mutation causing diseases including deletions, duplications and rearrangements. Most recently, retrotransposons were found to trigger neuronal diversity by inducing mosaicism in the brain. Finally, boosted during viral infections, Alu clearly interact with the innate immune system. The purpose of that review is to give a condensed overview of all these major findings that concern the fascinating physiology of Alu from their discovery up to the current knowledge.

## Background

Houck C et al.discovered and characterized the Alu repeats in 1979 [1]. Briefly, the authors isolated DNA from human placenta and radio-iodinated it. Using a DNA re-association kinetics technic called C_o_t, the DNA was denatured by boiling before partial renaturation at 60 °C. The remaining single stranded DNA was digested by S1 nuclease. The authors found a clear enrichment of 300 base pairs (bp) long DNA duplex. They wished to determine whether these repeated elements belong to one family of sequences. They purified these 300 bp repeats and digested it with restriction enzymes. *Alu*I from *Arthrobacter luteus* bacterium cuts AGCT and producing blunt ends in a methylation insensitivity manner. *Alu*I was able to produce two bands of 170 and 120 bp digesting “at least half” of these duplexes, therefore, the authors named these duplex “Alu” and conclude that they should belong to the same family of highly repeated sequences. They also reported that Alu represent 5% of the human genomic mass. Today, we know that multiple copies of Alu are dispersed into the human genome representing more than 10% of it (Table 1), whereas 99% of each Alu repeats consist of unique sequences [2]. The discovery of the Alu repeats raised questions concerning both a putative function and their origin.

**Table 1:** Representation of Alu sequences in the human genome

Alu number	Alu Gb	% of haploid genome	References
> 500,000	0.15	4.5	[3]
923,277	0.28	8.4	[2]
965,000	0.29	8.8	[4]
1,100,000	0.33	10	[5]
1,500,000	0.45	13.6	[6]

## Hypothetical origin

### Alu repeats derived from the 7SL RNA via a fossil Alu monomer (FAM)

Briefly, 7SL RNA (encoded in Human by the 299 bp RN7SL1 gene) is the RNA component of a universally conserved ribonucleoprotein complex, named the signal recognition particle (SRP). SRP is essential for translocation of secreted proteins. The cloning of the 7SL RNA highlights a high homology between the 5′ end of 7SL RNA and the Alu DNA repeats [7]. Later on, broader sequence comparison revealed that the 7SL RNA consists in an Alu monomeric sequence interrupted by a 155 bp long and 7SL RNA specific sequence [8]. That observation supports the hypothesis that an Alu monomer derived from a deletion of the central part of the 7SL RNA. Further, an 11 bp residual sequence (5′-TGTGAATAGCC-3′) of the 7SL gene localized specifically in the free right Alu monomer (FRAM) and not in the free left Alu monomer (FLAM). For that reason, geneticists thought that the FRAM should precede the FLAM. Phylogenic analyses were compatible with the hypothesis that a 7SL RNA gives rise first to a fossil Alu monomer (FAM) as a common ancestor for short interspersed nuclear elements (SINE). Then, the FAM gives rise to the primate-specific FRAM and FLAM before they fused together, giving rise to a dimeric mobile Alu [9]. Dimeric Alu propagate in the genome during 65 million years of evolution [10] representing today the most abundant repeats found in the human genome [11], see Table 1.

### Alu families

Alu repeats are subdivided in three families due to different consensus sequences and different times of appearance in the human lineage. Mutation densities were used to estimate the approximate age of each family [12]. These families were originally discovered by restriction digestion and named differently across the bibliography such as “PV”, “precise”, “major” [13], before a consensual nomenclature (“Y”, “S”, “J”) was adopted [14]. Alu families were further divided into subfamilies based on comparisons between aligned Alu sequences revealing high heterogeneity of certain positions (Table 2).

**Table 2:** Alu retro-transposition rates per births

Alu insertion per birth	References
1/200	[3]
1/40	[15]
1/20	[16]
1/21	[17]

The first 65 million years old AluJ family is the oldest one, encompass inactive Alu repeats, meaning these repeats lost retro-transposition potential due to the accumulation of mutations. The first subdivision thus segregates that AluJ family, more similar to 7SL RNA than the AluS family, itself subdivided afterwards [18]. The second AluS family is 30 million years old and still contains some active Alu. The third AluY family is the most active, aged between 2 and 4 million years old [19]. The Yb subfamily of the AluY family seems the most actively evolving nowadays [12]. Currently, more than 6000 Alu retrotransposons are active per human genome and belong to Y and S subfamilies [5].

### Alu retro-transposition

It is accepted today that the high number of Alu repeats has increased due to retrotransposition. Alu retrotransposition is a “copy and paste” process, altering the structure of the human genome, driving genomic variations and causing inheritable genetic diseases. Retrotransposition implies transcription, as well as an endonuclease activity to cleave the future integration site and a reverse transcriptase combined with an integrase activity to produce the cDNA and to insert it within a new site.

**Fig. 1:**
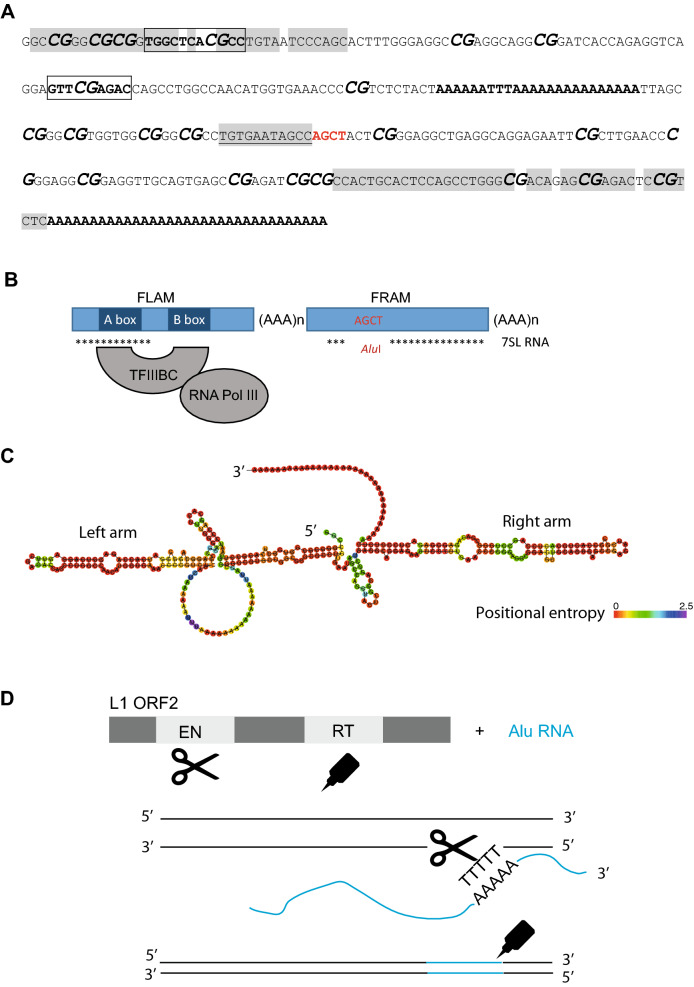
Alu illustrative sequence and retrotransposition. **A** A 328 pb long sequence representing an Alu that hits by blast at numerous positions in *Homo sapiens*, *Pan troglodytes* and *Gorilla gorilla* genomes. **AGCT**: *Alu*I restriction site characterizing the repeats as deriving from one family at their discovery giving them the name “Alu”. ***CG***: CpG sites in which the cytosines may be methylated appear in bold italic with an increased size of the font. Here 22 CpG sites are present in that Alu illustrative sequence. Highlighted in gray, homologous sequences with the RN7SL1 gene encoding 7SL RNA thought to be the origin of the Alu. The TGTGAATAGCC 11 bp residual sequence of the 7SL gene localized specifically in the free right Alu monomer (FRAM), showed both underlined and highlighted. Evolutionary, the FRAM precede the FLAM.  = A box,  = B box, both recognized by TFIIIBC and located in the first Alu monomer. These boxes promote transcription by RNA Pol III. Both contain a CpG site. **AAAAAATTTAAAAAAAAAAAAAAA:** the first poly-A tail separating both Alu monomers. **AAAAAAAAAAAAAAAAAAAAAAAAAAAAAAAAA:** the second poly-A tail which length may be proportional to transposition efficiency. **B** Transcription of Alu by RNA Pol III. TFIIIBC binds to A and B box in the FLAM of Alu. The stars represent the positions conserved between Alu and the 7SL RNA. **C** Predicted secondary structure of Alu RNA with the left and right arms as well as its 3′ poly-A tail. The structure was predicted using the Vienna RNA website [25]. Note that proteins bind to Alu RNA, not shown. **D** transposition of Alu initiated with an Alu RNA and the L1 ORF2. L1 ORF2 contains an endonuclease domain (EN) symbolized as scissors and a reverse transcriptase domain (RT) symbolized as a glue. EN cuts the DNA at T-rich sequence, allowing the binding of the Alu 3′ poly-A tail. Reverse transcription of Alu RNA to cDNA is done by RT. Note that the second DNA strand is cut and the second Alu cDNA strand is synthesized resulting in a new Alu element in the genome flanked by short direct repeats, not shown

Integration of Alu occurs in the canonical L1 endonuclease recognition sequence “TTAAAAA” [20]. An “A” box (TGGCTCACGCC) and a “B” box (GTTCGAGAC) are present within the internal promoter of numerous Alu repeats recognized by the TFIIIC and TFIIIB, triggering RNA Pol-III mediated transcription; see Fig. 1 [21, 22]. ORF2 protein encoded by another transposon named L1 carries both endonuclease and reverse transcriptase domains required for Alu retrotransposition [23]. Alu reverse transcription is initiated directly at the target locus after cleaving genomic DNA with target-primed reverse transcription (TPRT) or alternative mechanisms [24].

Functional study on Alu retrotransposition was limited since a French group specialized in the study of retro-transposon was able to generate a neo^tet^ construct into an Alu. First, they inserted the *Tetrahymena thermophila* 23S rRNA group I intron (tet) within a neomycin-containing cassette (neo). The tet intron is auto-catalytic, once transcribed, it can self-splice in vitro without the requirement of cellular components. In addition, the splicing out of the intron restores the neomycin-coding sequence. They introduced the construct within a vector itself transduced in infected cells some becoming resistant to the G418 antibiotic. That assay was able to recognize G418^R^ cells in which the splicing occurs [26].

In 2003, the same group introduced the neo^tet^ construct separately into both polyA regions of the Alu sequence located within the NF1 gene that was found in a patient suffering from neurofibromatosis. The neo^tet^ marked Alu was introduced thereafter between the enhancer and the transcription terminator of the Alu-like 7SL RNA. This marked Alu was then used to measure Alu mobilization. Indeed, the neo cassette becomes functional only after a cycle of retrotransposition, i.e. one cycle of transcription, reverse transcription and integration. Transposition was thus quantifiable by counting the number of G418^R^ clones. The assay demonstrates that the marked Alu was able to retro-transpose itself actively in the human genome when co-transduced with an expression vector for LINE proteins essentially ORF2. In addition, Alu insertions identified by invert PCR occur across different chromosomes and the presence of the second poly-A tail was required for Alu retro-transposition. Redundant sequences sandwiches the inserted Alu due to target site duplication (TSD). The authors demonstrated LINE-ORF2 mediated retro-transposition of marked Alu [27]. Thus, convincing results demonstrate that Alu retrotransposition is not autonomous: Alu parasite the L1 retro-transposition machinery to achieve their own replication. Therefore, Alu may be seen as a parasite’s parasite [28].

### Clinical consequences of ALU retrotransposition

Alu insertion may happen de novo. Depending on the insertion site, the mutation may affect human health. In 1991, Wallace et al. reports the first known de novo Alu insertion discovered by Sanger sequencing of PCR products in a patient affected by neurofibromatosis of type 1, a rare genetic disease with increased risk for tumor formation in the nervous tissues. The insertion occurred within an intron located between exon 5 and 6 of the NF1 gene. The authors thought that the Alu insertion altered the splice site by affecting the branch-point recognition process. One NF1 copy produced a shortened mature RNA lacking exon 6. That Alu insertion occurred de novo, it was absent in the DNA of both the mother and the father [29]. This Alu belongs to the youngest Y family and was functional for transposition as demonstrated decades after with the development of the Alu mobilization assay previously described. Two years after, a second de novo pathogenic Alu insertion was discovered in a patient suffering from severe haemophilia B. That time, the insertion of Alu interrupted the reading frame within the exon V of the Factor IX gene, resulting in a premature stop codon at glutamic acid 96 [30]. These two reports demonstrate together that insertions of Alu in either introns or exons may happen de novo and may cause diseases. The insertions most probably affected the germ cells, otherwise it would create mosaicism, as explained later.

In addition to pathogenic insertion by retrotransposition, Alu repeats concentrated in or near genes may trigger gene deletion or duplication by homologous recombination notably during crossing-over. In such cases, the retrotransposition of Alu is not required to cause the pathogenic events, but was required to create such Alu-rich regions in genes coding sequences. For example, Alu-mediated deletion by homologous recombination of the α-globin gene was implicated in α-thalassemia [31], whereas a duplication of a 14 kb sequence encompassing exons 2 through exon 8 in the LDL receptor gene produced a 50,000 Daltons larger LDL receptor in a patient suffering hypercholesterolaemia [32]. A worthy example concerns the abnormally high concentration of Alu repeats (41.5%) found within introns of the hyper-variable breast cancer 1 (BRCA1) locus. BRAC1 genotyping is used as a breast cancer predictor. Recently, the authors established that Alu may well be responsible for mediating BRCA1 gene rearrangements in patient’s tumors [33].

A reference review summarized the different clinical consequences of Alu and estimated that Alu contribute at least in 0.3% of all human genetic diseases [3]. Alu were responsible for insulin-resistant type II diabetes, Lesch–Nyhan syndrome, Tay–Sachs disease, complement component C3 deficiency, familial hypercholesterolaemia and several types of cancer including Ewing sarcoma, breast cancer and acute myelogenous leukaemia (see review). The contribution of Alu in the etiology of various human diseases is currently expanding. Alu retrotransposons contribute to at least 37 neurological and neurodegenerative diseases, mainly affecting mitochondrial functions [34]. Finally, the high pathogenic potential of Alu suggests the existence of cellular protective mechanisms.

### Epigenetic repression of Alu transposition

Epigenetic mechanisms silencing Alu to prevent retro-transposition were rapidly suspected to be required to protect the genome. It was estimated that 99% of Alu may be silenced resulting in 100 to 1000 cytoplasmic Alu transcripts per cell [35] from around 1 million of Alu DNA copies [5]. Thus, a very low level of Alu transcription occurs at less than 0.001 transcripts per Alu copy in a cell. Among the known epigenetic mechanisms, DNA methylation was rapidly suggested to prevent Alu transcription [36]. DNA methylation implies the addition of a methyl group to cytosine, located within CpG dyads in mammals. DNA methylation is tough to package Alu into an inactive chromatin structure denying access of essential transcription factors, notably TFIIIB, TFIIIC and RNA Pol III. Lack of Alu transcription should prevent their transposition. Numerous lines of evidence determined that DNA methylation indirectly controls Alu transpositions by silencing Alu transcription.

The first line of functional evidence derived from HeLa cells treated with 8 µM 5-azacytidine, an inhibitor of the DNA methyltransferases. The treatment resulted in hypo-methylation of Alu, detected by dot blot of DNA after digestion with the methylation sensitive BstUI restriction enzyme. In addition, increased Alu transcripts after the 5-azacytidine treatment was shown with primer extension and northern blot [35]. Finally, cDNA cloning and sequencing allowed the authors to conclude that all families of Alu were transcribed by Pol III In vivo after de-methylation induced by the 5-azacytldine treatment in the HeLa human cells.

The second line of evidence concerns the composition and the status of the CpG sites localizes within Alu. Approximately 20 to 24 CpG sites able to be methylated are present per 300 bp long Alu sequences. This represents a ninefold excess of CpG as compared with the genome. In fact, 23% of CpG in the genome are localized within an Alu (7 millions per 30 millions). Moreover, these CpG within Alu have been found heavily methylated in numerous studies (9), whereas hypo-methylation of Alu associates with cancers and tumors [37]. Interestingly, CpG residues in the youngest Alu subfamilies, which lost the retro-transposition activity, are largely methylated in vivo [13].

The third line of evidence concerns the study across organisms revealing that the organisms containing the CpG methylation machinery can tolerate genomic expansion driven by Alu. Interestingly, these organisms present a decreasing proportion of CpG dinucleotides over evolutionary time [38]. That paper suggests that methylation in transposons is a prerequisite for genomic expansion, whereas associated GC depletions occurs because the methylated cytosine had increased chance of being mutated into a thymine by uncorrected spontaneous deamination. Thus, during evolution, Alu integration followed by methylation may well produce GC poor extra-DNA. This GC poor extra-DNA seems to be used by the host for genomic regulatory purposes [38]. Indeed, the extra DNA can supply the host with new promoters, enhancers, insulators and may thus establish novel gene regulatory networks that may confer an evolutionary advantage in particular environmental conditions.

Methylation mediated repression of Alu was and remains consensually accepted until the two Varshney et al.publications on the modifications in histones in Alu using chromatin immunoprecipitations based techniques [39, 40]. The authors claim that methylation at lysine 9 of histone H3 rather than DNA methylation prevents Alu transcription, whereas methylation of Alu prevents translocation. The authors did not find elevation of Alu transcription after treating HeLa and fibroblast cells with 4 µM 5-azacytldine, a twofold decreased concentration as compared with the Liu et al. paper. Increased Alu transcription and RNA Pol III loading were observed after treating cells with a selective inhibitor of the SUV39 methyltransferases named chaetocin, the SUV39 being responsible for histone methylation. However, the most probable scenario seems that both DNA methylation and histone modification cooperate together to prevent both Alu transcription and transposition.

### Hypomethylated Alu in cancers

In theory, global Alu hypomethylation may increase the risk of cancer by promoting genomic instability, notably if Alu retrotransposition is poorly prevented. Another hypothesis implies Alu mediated expression of dominant oncogenes. In addition, as methylated CpG are more prone to mutation and concentrated within Alu, some of these mutations may also trigger cancers. In contrast, hypermethylated Alu located in the promoter of recessive tumor suppressors, which appeared enriched in Alu repeats [41], may also increase the risk of cancers, by interfering though silencing or even rearrangement, such as in the p53 [42], with an important mechanism of defense. Interestingly, the first human oncogene (human bladder carcinoma (EJ) oncogene) was discovered using an Alu marker-rescue experiment [43]. Today, the methylation status of Alu repeats was studied in various cancer types as a putative contributor to cancer etiologies. In a recent meta-analysis including 2719 cancer cases and 3018 controls, the conclusion was that hypomethylation of Alu occurs in carcinoma [44].

### Hypomethylated Alu in aging

Aging is a timeline natural process with increased cellular senescence, risks of degenerative diseases and deaths. Currently, aging at the cellular and organism levels may well be epigenetically encoded. Indeed, the Horwath epigenetic clock takes measures of DNA methylation at specific CpG sites to successfully predict ages [45], whereas the genomic hypomethylation hypothesis of aging postulates that the global level of DNA methylation decreased with ages [46]. It would be interesting to assess the proportion of CpG sites incorporated within the epigenetic clock that belongs to Alu repeats, whereas methylation of Alu repeats is already a good surrogate of the entire epigenome [47]. In that aging context, possible roles of human Alu elements in aging could be important [48]. Indeed, Alu are an endogenous source of genomic instability and thus may well promote contribute to lifespan variation. Epigenetic controls of Alu required enzymatic activities (DNMTs) whose efficiency may decrease during aging, notably for DNMT1 in aging fibroblasts [49].

### Alu and reprogramming

Reprogramming is a dynamic genome-wide erasure of DNA methylation marks followed by the establishment of new ones [50]. Reprogramming may theoretically trigger mobilization of all transposable elements that are usually kept silent inside compacted and methylated DNA such as L1-dependant Alu retrotransposons. Mobilization of Alu represents a high risk for the maintenance of the genomic integrity. Therefore, the epigenetic status of transposable elements as well as the identification of protecting mechanisms preventing detrimental transpositions of Alu during reprogramming are of high concern.

Naturally, reprogramming is linked to the sexual reproduction and occurs in two distinct “waves” affecting the germ cell lineage, conferring extensive developmental potential. The first reprogramming wave begins in the zygote, just after the fertilization and confers totipotency, meaning the ability to develop itself into all specialized cells forming an organism. The reprogrammed zygote gives rise to the pluripotent embryonic stem cells (ESC) able to differentiate into the three known germ layers named endoderm, mesoderm and ectoderm [51]. The second reprogramming wave occurs during the fetal life in the primordial germ cells (PGCs). In contrast to the zygote, PGCs remain highly specialized and unipotent, as being the progenitors of the gametes. However, the gametes present the potency to give rise to the next generation.

Artificial reprogramming occurs when biologists cultivate in vitro human ECS (hECS) or when differentiated cells are reprogramed by a pool of factors to produce human induced pluripotent stem cells (hiPSC). The purpose of the next chapters is to screen for known epigenetic status of the Alu and protective mechanisms against their activation in hiPSC and hECS as well as in the zygote and the PGCs.

### Epigenetic status of Alu during induced-pluripotency

In 2007, a scientific revolution happens when Takahashi et al. successfully induced hiPSC from adult human fibroblasts by retrovirus-mediated transfection of the transcription factors Oct3/4, Sox2, Klf4, and c-Myc [52]. In 2011, different searchers de-differentiated fibroblasts into hiPSC using the Takahashi method. These authors observed numerous genetic defaults affecting the hiPSC, somatic mutations [53], copy number variations [54, 55], tumorigenicity [56] and epigenetic abnormalities [57]. Some years later, three studies converged and reported activations of transposons during the induced reprogramming performed to produce iPSCs [58–60].

Klawitter et al.began by collecting hiPSC and their matched parental cells. RT-qPCR revealed increased transcription of L1 containing the essential components of the retro-transposition machinery used by Alu (ORF1 and ORF2), whereas the L1 promoter was de-methylated according to bisulfite DNA sequencing. Then, they applied retrotransposon capture sequencing (RC-seq) to map the genomic integration sites of retrotransposon insertions. The results revealed increased transposition events both after reprogramming in hiPSC and during culture of hESC with an average of 1411 new Alu insertion per sample analyzed. 83% of all transposons insertions not previously referenced involved Alu, among which 88% of Alu concern the AluYa5 subfamily. The authors defined de novo insertion as not found in the parental matched cells, not previously referenced and not found in an earlier hESC passage or in multiple hiPSC or hESC lines. Among all de novo insertions, PCR validates eight L1, seven Alu and two SVA. That study demonstrates L1 and Alu retro-transposition in hiPSC apparently triggered by the induced reprogramming [58].

Thus, artificial reprogramming producing iPSCs increased deleterious Alu transposition compromising the high expectation for regenerative medicine. Of note, the retrovirus that transduced the reprogramming factors may well affect the endogenous retrotransposition activity found in hiPSCs, as explained later. However, the increased transposition detected in the cells reprogrammed by the transduced factors triggers the question of how the cells protect themselves from transposition during reprogramming in vivo and naturally.

### Epigenetics of Alu and reprogramming the zygote

**Fig. 2:**
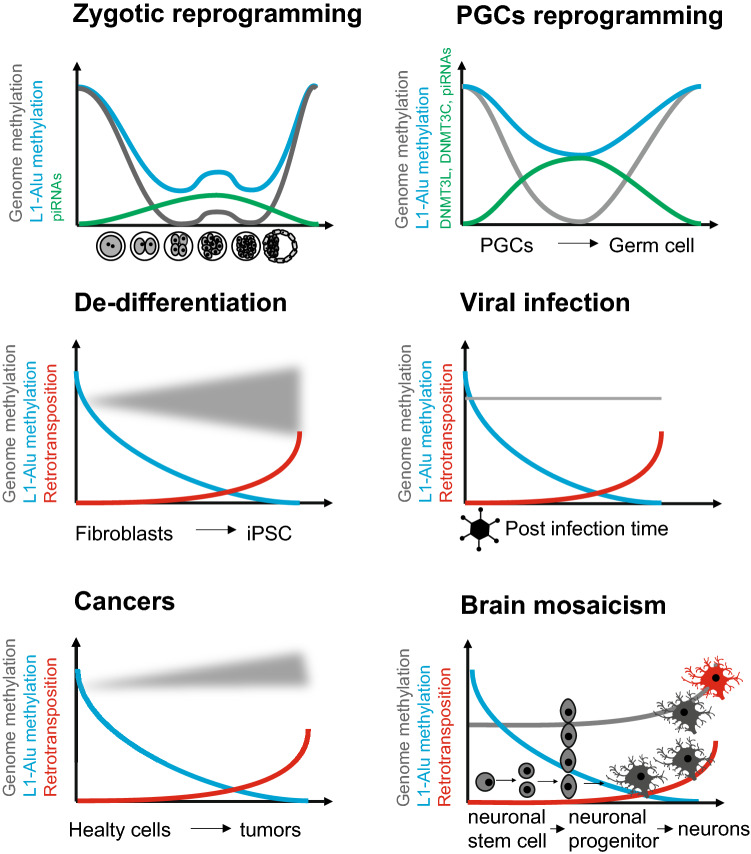
De-methylation and activation of L1-dependent Alu retrotransposon across reprogramming, de-differentiation, viral infections, cancers and brain mosaicism

The first wave of reprogramming starts in the zygote and ends once the blastocyst implants in the uterus. All cells composing the pre-implanted embryo that derived from the fertilized egg were reprogrammed. The methylation dynamic of Alu was indirectly studied during reprogramming of the zygote in human [61–63]. In those works, sperm and oocytes are collected from free-informed consent and healthy men and women donors. Then, intracytoplasmic sperm injection (ICSI) is performed on metaphase II oocytes to obtain zygotes. Some searchers also isolate both pronuclei in the zygote to study their demethylation processes separately. Morula are obtained at different stages in vitro (two cells, four cells, and so on) up to a blastocyst. Post-implanted embryos came from aborted fetuses. By following that research strategy, searchers were able to acquire human methylome data in all these successive cells, using bisulfite-sequencing techniques, producing datasets used to recapitulate DNA methylation dynamic during the zygote reprogramming process. Globally, data generated support enrichment of methylation in Alu and across the different development stages (Fig. 2).

**Fig. 3:**
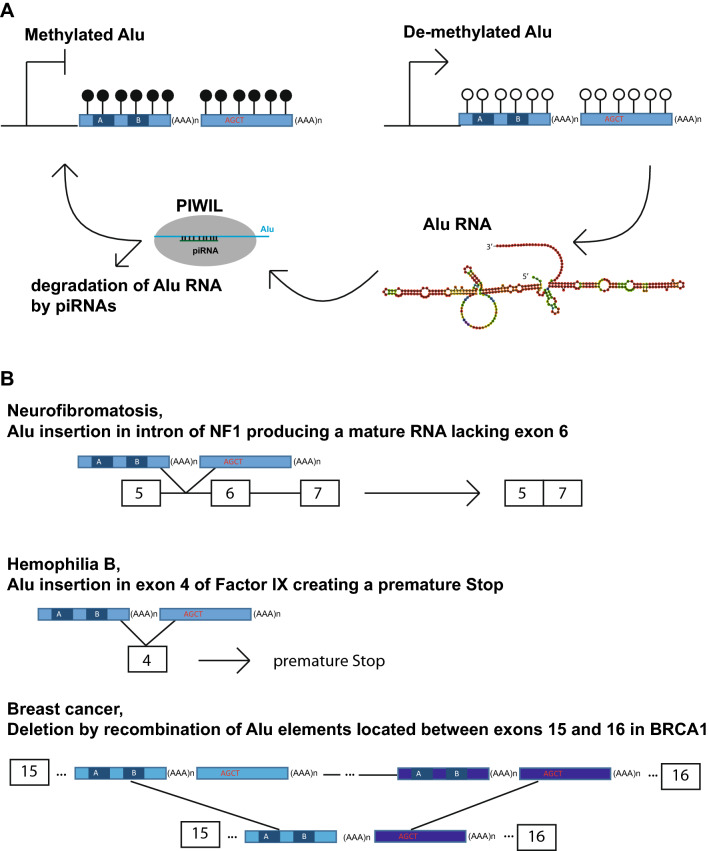
Mechanisms controlling Alu activities and examples of Alu in Human diseases. **a** Epigenetic and post-transcriptional mechanisms controlling Alu activity. DNA methylation prevent Alu transcription. In case of de-methylated Alu such as during reprograming, Alu RNAs could be post-transcriptionally controlled by P-element-induced wimpy testis (PIWI)-interacting RNAs (piRNAs). The piRNAs control retro-transposition by destroying the RNA encoded by Alu within the piRNA-induced silencing complexes (piRISC). In addition, piRNAs and may also help for de novo DNA methylation of Alu. **b** Role of Alu is developmental diseases. Three examples that are discussed in the text are schematized. First, an Alu insertion within an intron causing neurofibromatosis. Second, an Alu insertion within an exon causing Hemophilia. Third, rearrangement in an Alu rich region of BRCA1 important for breast cancers

As shown in the Supplemental Fig. 11 of the Smith et al. publication, three types of transposons (IAP, LINE containing the L1, and SINE containing Alu) most highly methylated in the sperm as compared with the zygote and the oocyte were more stable in term of methylation across cleavages in the morula [63]. This was consistent with the work of Guo et al. The later recorded complete human methylomes encompassing the embryonic reprogramming using both reduced representation bisulphite sequencing (RRBS) and whole-genome bisulphite sequencing (bs-seq) approaches [61]. Guo et al. results revealed global methylation of the DNA decreasing gradually from 41% genome-wide in the 2 cell morula to 29% in the inner cell mass (ICM) of the embryo, whereas in Alu sequences, the methylation levels obtained remains higher (around 50%) and more stable. This means that a bulk of methylation remains in the Alu repeats during the reprograming of the zygote, as observed in at least two independent works (Fig. 3).

In 2018, *Zhu *et al. provided similar tracing of the methylome changes across the first wave of zygote reprogramming at the single cell level, recapitulating more precisely the behavior of DNA methylation within Alu [62]. According to the results obtained, Alu strongly de-methylate from the late zygote to the two-cell stage as well as from the eight-cell to the morula stage. However, Alu and L1 enriched for de novo methylated CpG sites during both transitions from the early male pronuclear to the mid-pronuclear stage, as well as from the four to eight cells containing morula. According to the authors, de novo methylation favors Alu, most likely repressing their transcriptional activity to avoid mobilization and genome instability. Finally, the review of Greenberg claims that the highest retention of DNA methylation occurs in young and potentially active transposable elements, mentioning the SINE-VNTR-Alu (SVA) hominid-specific retrotransposon [64].

Thus, all the different studies converge for increased methylation once the embryos are implanted. The results obtained at the single cell resolution showed that the apparently stable methylation of Alu as seen in bulk samples and coinciding in time with a decreased methylation genome-wide in the developing zygote seems highly dynamic, and involves global demethylation and focal re-methylation. Further research on RNA-mediated silencing of Alu during the reprogramming of the zygote is strongly required. Indeed, germline specific RNAs may well control Alu transposition during precise time windows in which Alu are de-methylated. This could also explain focal re-methylation within Alu. That kind of mechanism was described for germline specific RNAs able to control retrotransposons during the reprogramming of the germ cells in plants, named the RNA-directed DNA methylation, silencing transposons, but the existence of that pathway in human seems unknown [65].

### Epigenetics status of Alu during the primordial germ cell reprogramming

The second reprogramming wave affects the primordial germ cells (PGC). PGCs are the precursor of the germ cell lineage and are reprogrammed when they colonized the gonads during the embryonic development. PGC reprogramming persists during the sexual differentiation process in the developing embryo. Re-methylation begins in the male-derived prospermatogonia or gonocytes and after birth in the female-derived growing oocytes. Note that most works performed on PGCs reprogramming were done in mouse lacking the dimeric primates specific Alu. Therefore, transposition of the knowledge from mouse to human is difficult.

In rodents PGCs, transposons bypass partly the reprogramming methylated by germline-cell specific DNMT3L and DNMT3C [66, 67]. The DNA methyl-transferases (DNMTs) consist of a family of enzymes able to transfer a methyl group from the S-Adenosyl Methionine (SAM) to the cytosine in the DNA, thus responsible for DNA methylation. The family contains the DNMT1 responsible for the maintenance of methylation across cell division, the DNMT3-A and -B that are able to methylate DNA de novo and both DNMT3L catalyzer, which is not able to methylate DNA alone but increased the activity of other DNMTs, as well as the germline-specific DNMT3C. DNMTs are essential for embryonic survival. DNMT3C protects male germ cells from transposon activity during reprogramming [66], whereas mice deleted for DNMT3L were sterile with adult males lacking spermatozoids as a consequence of a ‘catastrophic’ meiosis [67].

Importantly, in addition to DNA methylation, a subclass of the small non-coding RNAs also repress transpositions during PGCs reprogramming named the P-element-induced wimpy testis (PIWI)-interacting RNAs (piRNAs). The piRNAs are germline specific RNAs which main function is to control retro-transposition by destroying the RNA encoded by retrotransposons. The piRNAs may also help for de novo DNA methylation at some retrotransposon promoters [68]. A proof of principle in human cells cultivated in vitro revealed that the microprocessor (Drosha-DGCR8) negatively regulates LINE-1 and Alu retro-transposition [69].

### Alu and imprinting

In human, 30 to 200 genes may be imprinted (http://www.geneimprint.org). These imprinted genes present a somatic lifelong mono-allelic expression inversely correlated with a mono-allelic methylation status that depends on the parental origin of the alleles. The imprinting was thought to occur during gametogenesis, a period when both maternal and paternal genomes are clearly separated [70]. The silenced methylated allele should resist the zygotic reprogramming. In other words, paternal and maternal imprinted genes contain an allele deriving from either the sperm or the ovum pronuclei that bypassed the reprogramming and that are kept methylated and silenced lifelong. For example, PEG3, PEG10, PEG13, SNRPN, IGF2 and RB1 are imprinted paternally expressed genes, whereas MEG1, H19 and IGF2R are imprinted and maternally expressed genes.

The question emerged whether Alu and other transposons are also able to bypass reprogramming by using the imprinting mechanism. In the single cells methylome study of Zhu et al., differentially methylated regions (DMRs) were analyzed between oocytes and sperm [62]. The authors reported that all “31 known imprinting controls regions (ICR)” were included among these DMRs. When analyzing the distribution of these DMRs in the genome, they found strong enrichment in Alu elements. However, when John M. Greally studied the genomic composition in imprinted versus non-imprinted loci, he reported that imprinted regions lack Alu [71]. Thus, both parental gametes strongly differ in term of DNA methylation in both imprinted loci and Alu repeats without co-localization between them. Paradoxically, some imprinted regions were reported to be derived from previous transposition events, whereas genomic imprinting may have evolved from a defense mechanism against transposable elements depending on DNA methylation established in germ cells [72].

### Alu epigenetics and human infertility

The epigenetics control of Alu during both reprogramming waves and Alu involvement in the imprinting process are essential for the proper development of both the germ cells and the zygote. Thus, epigenetic defaults in Alu could explain at least some cases of infertility. Infertility in human was arbitrary defined by the world health organization (WHO) as the absence of pregnancy after one year of regular unprotected sexual intercourse in a couple. Even if arbitrary, the definition influences the access to assisted reproductive technologies (ART) for infertile couples. Epigenetics of Alu in the germ cells seems of high concern to understand the etiology of unexplained infertility [73], but also in child conceived with ART, as the incidence of rare imprinting disorders was increased in ART conceived children [74]. Currently, Alu methylation in germ cells of infertile men is expanding, but remains rarely, if not at all studied in the oocytes of infertile women. In general, the contribution of Alu in both men and women infertility remain largely understudied.

In an ongoing collaborative and unpublished study on DNA methylation in sperm of infertile men, for which half present normal sperm parameters, we recently identified demethylation of Alu by using a genome-wide approach and are actually assessing to replicate these results. These results were at the origin of my interest in the study of Alu. Urdinguio et al. reported compatible results: decreased cytosine methylation in Alu, notably the Yb8 in their work, in the sperm of men suffering from unexplained infertility [75]. In addition, Alu may also probably contribute to female infertility. One line of evidence concern NLRP7. Apparently, the correct gene copy of NLRP7 is required for the female reproductive contribution. Indeed, large homozygous deletion mutations identified in NLRP7 were responsible for deficient pregnancies and reproductive wastage [76], whereas the breakpoints are located within Alu repeats [77].

### Alu and mosaicism

Mosaicism implies the existence of two or more cells with different genotypes present within one individual and deriving from the same zygote. Alu retro-transposition affecting some embryonic cells may create mosaicism in the organism. The early embryo is the primary niche for the accumulation of new retrotransposition. Of importance, somatic mosaicism created by retrotransposition should not be heritable in contrast to retrotransposition affecting the germline. Pathogenic mosaicism caused by Alu retrotransposition in Human has already been reported [78]. In that work, multiple complex rearrangement sequences encompassing exon 1 of GAN were described for a patient suffering Giant axonal neuropathy. The patient’s genome(s) was analyzed by quantitative real-time PCR and breakpoint DNA sequencing. The authors identified micro-homology of Alu sequences at the breakpoint suggesting Alu mediated recombination [78].

However, human Alu mediated mosaicism remain poorly studied as compared with mosaicism involving L1 retrotransposition in rodents, especially in neuronal cells [79]. The following key biological aspects of mosaicism induced by retro-transposition strongly required to be confirmed in the humans Alu context. First, retrotransposons can mobilize both during embryogenesis as well as during division in the neuronal lineage causing somatic genome mosaicism. Second, the neuronal progenitor cells and post-mitotic neurons accommodate retrotransposition in contrast to other cell lineages. Finally, retrotransposition clearly occurs in the brain and creates neuronal diversity.

Today, we believe that the brain is mosaic [80]. The brain contains approximately 80 billion neurons derived from a colossal number of progenitor’s division. Thus, even if rare, retro-transpositions or wrongly repaired DNA errors could statically occur during that enormous amount of replication in neuronal progenitors, and are thought to participate in the brain mosaicism. Supporting that hypothesis, endogenous retro-elements have been used to successfully trace numerous lineage and sub-lineages of neuronal cells by Evrony et al., using whole-genome sequencing at the single neuron resolution [81]. The profiling of L1-transposon insertion suggested that 0.2 to 1 L1 insertion may occur per “neuronal genome” [80]. Actually, new evidences suggest that pathogenic transposition contributes to a broad range of neurological diseases which etiology remains poorly understood [82].

### Viral contribution to Alu transposition boom

Evidences suggest that Alu may also have originated from reverse transcribed 7SL RNA in a viral infected host. A first evidence supporting a viral origin of Alu concerns the detection of reverse transcription of 7SL RNA in a quail cell line infected by a deficient avian Rous sarcoma virus [83]. The authors demonstrated that 7SL RNA may serve as a template for reverse transcription in the virion with transfer RNA (tRNA) used as primer and resulting in a cDNA of 135 bp compatible in size with an Alu monomer. Unfortunately, the cDNA was not sequenced. Interestingly, the host 7SL RNA increased in cells infected by virus, later quantified approximately at 12 copy per retroviral particles [84].

The viral origin of Alu seems difficult to study, but recent evidences emerge reporting increased Alu retrotransposition in viral infected cells. One report experimentally demonstrates increased Alu transcription, transposition and copy in primary CD4 + cells infected by HIV-1 [85]. In 1995, Russanova et al.isolated nucleus of HeLa cells infected with adenovirus at 25, 50 and 150 plaque-forming units (PFU) compared with nuclei of uninfected cells. They developed an original method combining nuclear run-on and RNase H assay to assess how Alu are expressed. Their results revealed that Alu elements were masked from the Pol III transcriptional machinery in the nuclei of uninfected cells, whereas the Pol III machinery actively transcribed Alu in infected cell nuclei. The authors concluded that Alu repeats are efficiently sequestered by chromatin proteins and that adenovirus infection partially overrides that repressive mechanism [86]. Thus, the contribution of different virus boosting Alu transposition during evolution is credible.

### Innate immune response and Alu

The innate immune system is an evolutionary conserved host defense system acting against retrovirus, but also against the activation of retrotransposon [87]. In contrast to a retrovirus, which assembles itself and buds out the infected cell before infecting other cells, Alu and other retrotransposons stay within the host cell. However, Alu and other retrotransposons are present as DNA sequences in all human cell’s and may add a new DNA copy of them transmissible to all cells constituting the next generation in case the retro-transposition affects the germline. Once retrotransposons such as Alu are expressed in a cell, for example, during a viral infection [86], various nucleic acid forms of Alu may theoretically appear, namely (1) single stranded RNA (ssRNS), (2) RNA:RNA duplex or double stranded RNA (dsRNA), (3) RNA:DNA heteroduplex, and (4) DNA:DNA duplex or double stranded DNA (dsDNA). Apparently, both RNA:RNA and DNA:DNA Alu duplex may appear in the cytoplasm as intermediates and may be sensed by host cell derived molecular sensors. The melanoma differentiation-associated protein 5 (MDA5) detects the presence of RNA:RNA Alu [88]. It remains unknown if the cyclic GMP-AMP synthase (cGAS) detects DNA:DNA Alu [89]. The innate immune system should tolerate a basal presence of Alu or it will trigger targeted nucleic acid destruction. In addition, the innate immune system may produce cytokines and inflammation, useful to fight the virus, but useless in case of within-cell restricted retrotransposon, excepted in the case of a transposon-induced cancer. In fact, the immune system has to tolerate domesticated Alu, balancing between recognition of self and activation of innate immunity. Indeed, if the innate immune system acts too strongly to Alu, it may eventually give rise to autoimmune disorders [90].

Concerning nucleic acid forms, first, ssRNA Alu is produced by Pol III-dependent transcription [21] and is destroyed by Dicer1 [91]. In case of geographic atrophy, a common cause of blindness in industrialized countries, Dicer is deficient and increased ssRNA Alu toxicity is detectable in the eyes [91]. Alu RNA mediated toxicity affects more precisely cells constituting the retinal pigment epithelium [92]. Second, Alu RNA–DNA heteroduplex may be produced during reverse transcription as a common feature of retrotransposons, as well as DNA:DNA duplex required for the integration of a new copy of the transposon [87]. In addition to these forms, Alu RNA:RNA duplex has been identified [88] deriving from sense combined with antisense transcription [87]. The most convincing evidence supporting the involvement of Alu in innate immune response came from a study by Ahmad et al*.* on dsRNA sensor MDA5 in Aicardi-Goutières syndrome (AGS), an inherited encephalopathy [88]. Alu RNA:RNA hybrids formed by inverted Alu activate the MDA5 mutated gene found in AGS but not wild-type MDA5. The authors reported that in AGS, Alu RNA:RNA duplex was the primary ligands triggering aberrant MDA5-mediated antiviral signaling [88]. MDA5 was known as a cytosolic innate immune sensor of dsRNA generated during viral replication [93].

One report discovered that Alu may modulate autoimmunity in case of systemic lupus erythematosus (SLE). SLE is an autoimmune disease caused by the production of autoantibodies targeting the protein Ro60. When Hung et al. aim to identify RNAs bound to Ro60 using individual nucleotide crosslinking and immunoprecipitation (iCLIP) followed by sequencing, they discovered Alu RNA bounded to Ro60 [94]. These Alu RNAs were increased in the blood of patients suffering SLE as compared to controls. Alu RNAs and interferon-regulated genes increased also in the absence of the Ro60 gene in cell culture, as well as in the presence of interferon alpha (IFNα). Thus, Alu RNA levels were linked to the Ro60 autoantibody responses in SLE and may probably contribute to human autoimmunity. Finally, according to Herbert, Alu may regulate inflammation based on their potential to form various conformations, enabling a rapid reprogramming of cellular pathways. Alu may induce so called non-B-DNA conformations named Z-DNA, triplexes and quadruplexes. These particular conformations modified the way the genome is expressed [95].

## Conclusion

Alu retrotransposons are involved in fundamental biological processes (Fig. 2). In addition, Alu may contribute to numerous genetic diseases with apparently unexplained etiologies. The clinical impact of Alu is probably underestimated, as Alu remain badly understood in the medical genetic and clinical context. The physiology of Alu retrotransposition remains to be better studied, as the mechanism simplified in the present review is still not completely understood at the molecular level. Numerous questions remain open concerning Alu. During reprogramming, it would be interesting to know if RNA interference controls Alu during precise time windows in which Alu are de-methylated in the zygote. The interaction between piRNAs and de novo methylation in the germ cells is possible but unknown in human. That putative interaction could provide a better understanding of how reprogramming did not allow massive retrotransposition. Concerning the involvement of Alu in infertility, it would be of great interest to study the epigenetic status of the Alu that are located in the NLRP7 locus in the oocytes of woman suffering miscarriages, as well as the methylation of Alu in the sperm of men suffering unexplained infertility. How to explain the paradox that imprinted regions significantly lacks Alu repeats, whereas imprinting may be derived from previous transposition events? Considering that virus may “wake up” Alu, what would be the impact of the actual pandemic coronavirus on the Alu mediated evolution of our genomes? In the innate immunity, are Alu DNA:DNA detected in the cytoplasm by cGAS? Does Alu retrotransposition occur in the neuronal lineage creating mosaicism as found for L1 retrotransposition? Thus, numerous studies still may be conducted on the fascinating L1-dependant and Pol III transcribed Alu retrotransposon.

## Abbreviations

AGS: Aicardi-Goutières syndrome
ART: Assisted reproductive technologies
BRAC1: Breast cancer 1
cGAS: Cyclic GMP-AMP synthase
DMRs: Differentially methylated regions
DNMTs: DNA methyl-transferases
dsDNA: Double stranded DNA
dsRNA: Double stranded RNA
ESC: Embryonic stem cells
FAM: Fossil Alu monomer (FAM)
FLAM: Free left Alu monomer
FRAM: Free right Alu monomer
hiPSC: Human induced pluripotent stem cells
iCLIP: Individual nucleotide crosslinking and immunoprecipitation
ICR: Imprinting controls regions
ICSC: Intracytoplasmic sperm injection
IFNα: Interferon alpha
MDA5: Melanoma differentiation-associated protein 5
MEG: Maternally expressed gene
PEG: Paternally expressed gene
PFU: Plaque-forming units
PGCs: Primordial germ cells
piRNAs: P-element-induced wimpy testis (PIWI)-interacting RNAs
RC-seq: Retrotransposon capture sequencing
SAM: S-Adenosyl Methionine
SINE: Short interspersed nuclear elements
SLE: Systemic lupus erythematosus
SRP: Signal recognition particle
ssRNS: Single stranded RNA
SVA: SINE-VNTR-Alu
TPRT: Target-primed reverse transcription
TSD: Target site duplication
